# A Highly Predictive MicroRNA Panel for Determining Delayed Cerebral Vasospasm Risk Following Aneurysmal Subarachnoid Hemorrhage

**DOI:** 10.3389/fmolb.2021.657258

**Published:** 2021-05-14

**Authors:** Wang-Xia Wang, Joe E. Springer, Kevin Xie, David W. Fardo, Kevin W. Hatton

**Affiliations:** ^1^Sanders Brown Center on Aging, University of Kentucky, Lexington, KY, United States; ^2^Spinal Cord and Brain Injury Research Center, University of Kentucky, Lexington, KY, United States; ^3^Pathology & Laboratory Medicine, University of Kentucky, Lexington, KY, United States; ^4^Neuroscience, University of Kentucky, Lexington, KY, United States; ^5^Biostatistics, University of Kentucky, Lexington, KY, United States; ^6^Anesthesiology Critical Care Medicine, University of Kentucky, Lexington, KY, United States

**Keywords:** microRNA, biomarker, aneurysmal subarachnoid hemorrhage, delayed cerebral vasospasm, cerebrospinal fluid, plasma

## Abstract

Approximately one-third of aneurysmal subarachnoid hemorrhage (aSAH) patients develop delayed cerebral vasospasm (DCV) 3–10 days after aneurysm rupture resulting in additional, permanent neurologic disability. Currently, no validated biomarker is available to determine the risk of DCV in aSAH patients. MicroRNAs (miRNAs) have been implicated in virtually all human diseases, including aSAH, and are found in extracellular biofluids including plasma and cerebrospinal fluid (CSF). We used a custom designed TaqMan Low Density Array miRNA panel to examine the levels of 47 selected brain and vasculature injury related miRNAs in CSF and plasma specimens collected from 31 patients with or without DCV at 3 and 7 days after aSAH, as well as from eight healthy controls. The analysis of the first 18-patient cohort revealed a striking differential expression pattern of the selected miRNAs in CSF and plasma of aSAH patients with DCV from those without DCV. Importantly, this differential expression was observed at the early time point (3 days after aSAH), before DCV event occurs. Seven miRNAs were identified as reliable DCV risk predictors along with a prediction model constructed based on an array of additional 19 miRNAs on the panel. These chosen miRNAs were then used to predict the risk of DCV in a separate, testing cohort of 15 patients. The accuracy of DCV risk prediction in the testing cohort reached 87%. The study demonstrates that our novel designed miRNA panel is an effective predictor of DCV risk and has strong applications in clinical management of aSAH patients.

## Introduction

Aneurysmal subarachnoid hemorrhage (aSAH) is a catastrophic event that occurs when an intracranial arterial aneurysm ruptures, resulting in the release of oxygenated blood into the subarachnoid space (Dority and Oldham, 2016). Delayed cerebral vasospasm (DCV) has long been considered the most devastating acute complication following aSAH (Janjua and Mayer, 2003; Al-Mufti et al., 2017; Brami et al., 2020). Approximately 30% of aSAH patients develop DCV (Janjua and Mayer, 2003), which commonly occurs during 3–10 days after aneurysm rupture, requiring frequent neurologic monitoring and rapid, intensive interventions to minimize further injury and disability. The pathological events contributing to DCV are poorly understood but appear to include calcium dependent and independent vasoconstriction, oxidative stress, endothelial dysfunction, inflammatory responses, apoptosis, autophagy, and altered gene expression (Macdonald and Weir, 1991; Dietrich and Dacey, 2000; Kolias et al., 2009; Carr et al., 2013; Eisenhut, 2014; Miller et al., 2014; Chou, 2018). Predicting DCV risk before it becomes clinically symptomatic is crucial to limiting further injury. Unfortunately, no effective biomarker exists to predict DCV at a time point when neurologic injury can be prevented (Mocco et al., 2006; Etminan et al., 2011). At this time, clinical management includes a “wait and see” approach. Therefore, there is an urgent need to identify biomarkers that determine DCV risk/susceptibility in order to implement preventive or management strategies.

MicroRNAs (miRNAs) are small, non-coding, regulatory RNAs (Bartel, 2004; Huntzinger and Izaurralde, 2011). MiRNAs regulate all aspects of cellular function, and disruption of miRNA activity contributes to many disease states including neurological diseases such as Alzheimer’s disease, stroke, and traumatic brain injury (Wang et al., 2008, 2010, 2015; Liu and Xu, 2011; Tan et al., 2011; Koutsis et al., 2013; Hill and Lukiw, 2016). Several studies have demonstrated changes in miRNA levels in cerebral arteries following aSAH and DCV (Vikman et al., 2006; Muller et al., 2015; Li et al., 2018). For example, over 150 miRNAs were differentially expressed in the aneurysmal tissue when compared to normal arteries (Liu et al., 2014). MiRNAs are also recognized as powerful regulators of CNS inflammatory responses (O’connell et al., 2012), including that occurs following stroke and aSAH (Khoshnam et al., 2017; Lopes et al., 2018). Their functional role and the fact that miRNA can be secreted into extracellular fluids make them attractive candidates as biomarkers. Moreover, miRNAs in biofluids are highly stable at room temperature, resistant to RNase activity (Mraz et al., 2009) and relatively easy to detect and quantify (Etheridge et al., 2011). In this study, we explore the potential of using cerebral spinal fluid (CSF) and plasma miRNAs as biomarkers to predict DCV events in aSAH patients.

## Materials and Methods

### Patient Cohorts

All procedures and protocols related to the human participants and collections of specimens were approved by the University of Kentucky Institutional Review Board (IRB) that are in compliance with the 1964 Helsinki declaration and its later amendments. Following the approved IRB protocol (# 55914), all patients admitted to the University of Kentucky Chandler Medical Center (UKCMC) were screened for inclusion in this study. Inclusion criteria included being 18 years of age or older, radiographically evidence of aSAH, a Modified Fischer Score > 3 (Frontera et al., 2006), and a ventriculostomy (EVD: external ventricular drain) being placed prior to post-bleed-day 3 (PBD3). Patients were excluded if they arrived at the hospital in a non-survivable condition or were likely to die prior to PBD14 as determined by the attending physicians. Patients also were excluded if they had a history of systemic inflammatory disease or chronically dosed with a biologic inflammatory modulator. Finally, patients were excluded if a legally authorized representative (LAR) could not be located by PBD3 or who refused (or their LAR refused) consent.

During hospitalization, all patients were treated according to local standard of care. Patients were considered to have DCV if, during their hospitalization, the daily transcranial Doppler (TCD) monitoring demonstrated a mean flow velocity > 120 cm/sec in either middle cerebral artery (MCA) and a calculated Lindegaard ratio >3.0 (Kirsch et al., 2013).

### Cell-Free CSF and Plasma Sample Collection

After written consent was obtained, CSF and plasma were collected at PBD 3, 5, 7, and 10 using a standard protocol. CSF was collected directly from the EVD drainage tubing. Collected samples were centrifuged at 3,000 × g for 5 min and the supernatant aliquoted into 250-μl aliquots and frozen at -80 C until further analysis. The miRNA analysis was performed only from the specimens collected at PBD3 and PBD7.

In addition, frozen plasma and CSF samples from several de-identified cases were gifted from the University of Pennsylvania (UPENN). This data set and biofluids had already been collected and no information on data collection or sample preparation were available. Therefore, they only were used in testing prediction model.

Finally, CSF and plasma specimens from eight healthy persons who had no clinically manifest neurological disease/diagnosis were procured from the University of Kentucky Alzheimer’s Disease Center (UK-ADC) biobank.

### Experimental Design

**FIGURE 1:**
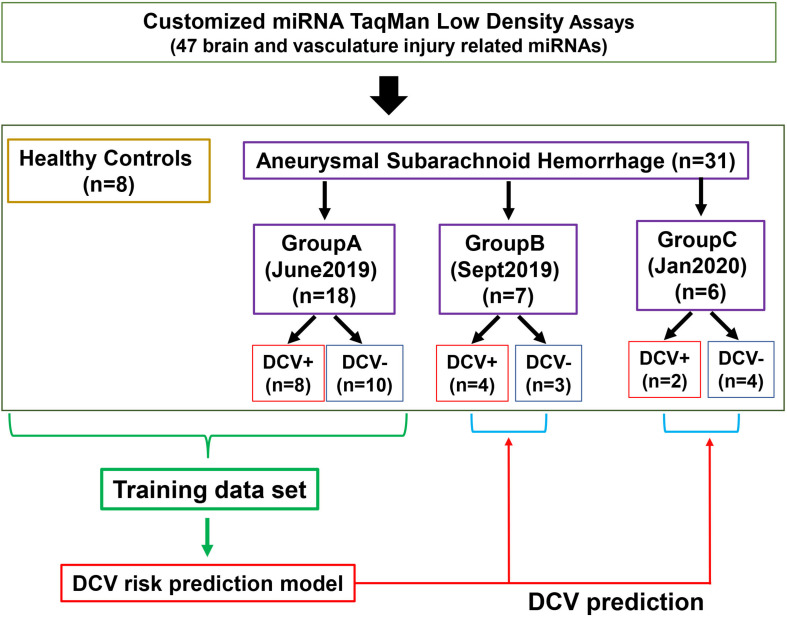
Flowchart of patient groups and sample analysis. DCV: delayed cerebral vasospasm.

A total of 39 patients and controls were analyzed in three separated groups according to the availability of the specimens at the time of analysis (Figure 1). Group A was analyzed in June 2019 and included eighteen aSAH patients (10 DCV-, 8 DCV+) from UKCMC and 8 healthy controls (HCs). Group B was analyzed in September 2019 and included nine aSAH patients including two experimental duplicates from Group A, three additional cases from UKCMC, and four cases from UPENN. Group C was analyzed in January 2020 and included six aSAH patients from UKCMC. The data from Group A was used as a training set to build a risk prediction model for testing the discrimination ability of the miRNA panel in Group B and Group C. Differences in demographics and clinical observations were evaluated using Fisher’s exact test.

A blinding procedure was implemented in this project. The physicians who diagnosed and treated the patient, and those that collected and aliquoted the specimens did not perform the miRNA analysis. Likewise, miRNA analyses were performed by personnel blinded to patient data and clinical outcome. Clinical and laboratory data were only unblinded after all data were acquired. The statisticians who constructed the prediction models were not involved in either the sample collection or miRNA analysis.

### Design of a Customized Brain and Vasculature Injury Related miRNA Panel

A customized panel containing miRNAs associated with brain and vasculature injury was designed as described previously (Wang et al., 2017) and used to generate a TaqMan^®^ miRNA RT-qPCR low density array (TLDA, Thermo Fisher Scientific, Waltham, MA, United States) that can simultaneously analyze 48 miRNAs in 8 independent samples.

### RNA Isolation From CSF and Plasma

CSF and plasma total RNA were isolated using miRNeasy Serum/Plasma Advanced Kit (Cat #: 217204, Qiagen, Hilden, Germany) following manufacturer’s protocol. RNA was eluted with 30 μl of nuclease-free water containing 0.5 U/μl RNAsin (RNase inhibitor, Cat #: N2115, Promega, Madison, WI, United States).

### RT-qPCR Using Customized TaqMan^®^ Low-Density Array (TLDA)

MiRNA reverse transcription and real-time PCR detection procedures were performed following the manufacturer’s instruction and are also described in detail in prior publications (Wang et al., 2014, 2017).

### TLDA Data Processing

Quantitative real-time PCR run files were imported to and analyzed using Thermo Fisher Cloud Software—Relative Quantification. The relative threshold cycle (C_rt_) method (Applied Biosystems, 2016) was applied to acquire the quantitative C_rt_ values from all runs in each experiment. C_rt_ values that failed software-defined QC and/or values equal to or more than 35 were considered as undetectable and excluded from further analysis. Furthermore, a miRNA would be removed from analysis if it was not detected in one third of tested samples. The TLDA consisted of specific selected miRNAs and an internal normalizer could not be identified. Therefore, the Global Mean Normalization method (Mestdagh et al., 2009) was used to normalize the TLDA data. Raw C_rt_ values and the geometric mean of a given sample was used to obtain ΔC_rt_ values calculated as ΔC_rt_ = C_rt__–__target_ - C_rt__–__geomean_ for each miRNA. The time for processing 2–8 samples from RNA isolation to data analysis is around 6–8 h (Supplementary Figure 1).

### Statistical Analysis

GraphPad Prism 8.0.2 (San Diego, CA, United States) and R (version 4.0.2) (R Core Team, 2020) were used for statistical analyses. Student’s *t*-tests (2-tailed) were used to evaluate mean differences for individual miRNAs. A 95% confidence interval with *p* < 0.05 being considered statistically significant. The receiver operating characteristics (ROC) were generated to evaluate DCV discrimination ability of individual miRNAs.

### Construction of DCV Prediction Model

A DCV prediction model was constructed using the Group A CSF/PBD3 dataset. The model used the 26 miRNAs (“variables,” Supplementary File 1) that did not have any missing data across all 3 patient groups. Among the 26 miRNAs, the expression levels of 7 miRNAs exhibited perfect discrimination between DCV+ and DCV- patient groups in the Group A dataset (Supplementary Figure 2 and Supplementary File 1). These seven miRNAs each served as individual predictors in a consensus predictive tool. The remaining 19 miRNAs were used to generate a decision tree using the Recursive partitioning and regression trees (Therneau and Atkinson, 2019) (Rpart) algorithm in R.

## Results

### Demographic and Pathological Data of Study Cohorts

From July 2018 to December 2019, 77 patients were admitted to UKCMC with aSAH, of these, 27 were included in this study. There were no significant differences between the patients with DCV (DCV+) and patients without DCV (DCV-) based on age, gender, height, weight, body mass index, ethnic origin (Table 1). Furthermore, no significant differences were observed between the two study groups in the classification of the severity of subarachnoid hemorrhage (Hunt Hess score, WFNS score, and modified Fischer score), location of the aneurysm, or the type of aneurysm obliteration. The average age of the HC group was older than that of aSAH patients, however, we were primarily interested in the comparison between DCV+ and DCV- patients and the HC group served as a secondary reference. Therefore, the age difference was not considered a confounding factor.

**TABLE 1:** Demographic and statistics of the study population.

	**Healthy control *N* = 8**	**DCV *N* = 12**	**No DCV *N* = 17**	***p*-value (DCV vs. No DCV)**
Age (years)	74.3 ± 7.0	59.3 ± 18.2	59.4 ± 12.3	0.99
Gender (male)	3 (37.5%)	3 (25.0%)	9 (52.9%)	0.25
Height (cm)	N.D.	166.9 ± 11.6	171.8 ± 10.1	0.24
Weight (kg)	N.D.	97.1 ± 27.4	92.7 ± 26.8	0.67
Body mass index (kg/m^2^)	N.D.	34.6 ± 7.7	30.9 ± 6.6	0.18
Ethnic origin (White/Caucasian)	8 (100%)	11 (91.7%)	15 (88.2%)	1.00
Hunt Hess score (3–5)	N/A	8 (66.7%)	10 (58.8%)	0.72
WFNS score (3–5)	N/A	6 (50.0%)	5 (29.4%)	0.44
Modified Fischer score (4)	N/A	6 (50.0%)	7 (41.1%)	0.71
Aneurysm located (yes)	N/A	12 (100%)	13 (76.5%)	0.12
Aneurysm location (anterior)	N/A	7 (58.3%)	7 (53.9%)	0.46
Aneurysm obliteration type (coiled)	N/A	11 (91.7%)	13 (100%)	0.37

**Baseline demographics of aneurysmal subarachnoid hemorrhage (aSAH) patients from UKCMC grouped based on whether or not they developed delayed cerebral vasospasm (DCV) during hospitalization. Continuous variables are described as mean ± SD with differences evaluated by an unpaired t-test. Categorical variables are described as N and% with differences evaluated by Fisher’s exact test. N.D., no data available; N/A, not applicable.**

### Design and Performance of the Customized Brain and Vasculature Injury Related miRNA Panel

We developed a customized miRNA panel consisting of 47 miRNAs relevant to brain and vasculature injury events (Table 2). MiRNAs that met any of following criteria were selected: (1) strong association with CNS injury or cerebrovascular damage, either identified in our prior studies (Wang et al., 2015, 2017) or reported in the literature; (2) implication in previous biomarker studies related to CNS injury (TBI, SCI, or neurodegeneration) or stroke; (3) potential data normalizer; (4) is detectable in CSF and plasma. The panel contained one additional rodent specific miRNA and the manufacturer’s mandatory control U6, both of which were excluded from analysis. CSF and plasma miRNA from a total of 31 aSAH patients (27 patients from UKCMC and 4 from UPENN) and 8 HCs were included in the TLDA analysis (Figure 1).

**TABLE 2:** Selection of brain- and vasculature injury related miRNAs.

**Assay ID**	**miRNA^a^**	**Sequence**	**Relevance to aSAH and cerebral vasculature**
000377	hsa-let-7a-5p^b^	UGAGGUAGUAGGUUGUAUAGUU	Inflammatory/immune response
002619	hsa-let-7b-5p^b^	UGAGGUAGUAGGUUGUGUGGUU	Angiogenesis/inflammatory and immune response
000379	hsa-let-7c-5p^b^	UGAGGUAGUAGGUUGUAUGGUU	Inflammatory/immune response
000439	hsa-miR-103a-3p^b^	AGCAGCAUUGUACAGGGCUAUGA	Cell migration/wound healing
000443	hsa-miR-107^b^	AGCAGCAUUGUACAGGGCUAUCA	Inflammatory response/cell migration/wound healing
000449	hsa-miR-125b-5p^b^	UCCCUGAGACCCUAACUUGUGA	Cell proliferation/neuronal Integrity
002884	hsa-miR-1274b^c^	UCCCUGUUCGGGCGCCA	Potential normalizer (CSF)
002861	hsa-miR-1298-5p^b^	UUCAUUCGGCUGUCCAGAUGUA	Neural regeneration
000457	hsa-miR-132-3p^b^	UAACAGUCUACAGCCAUGGUCG	Vascular angiogenesis
000464	hsa-miR-142-3p^b^	UGUAGUGUUUCCUACUUUAUGGA	Inflammatory and immune response
002248	hsa-miR-142-5p	CAUAAAGUAGAAAGCACUACU	Inflammatory and immune response
002676	hsa-miR-144-3p^b^	UACAGUAUAGAUGAUGUACU	Proliferation/apoptosis/oxidative stress
000468	hsa-miR-146a-5p^b^	UGAGAACUGAAUUCCAUGGGUU	Inflammatory and immune response
001097	hsa-miR-146b-5p	UGAGAACUGAAUUCCAUAGGCU	Inflammatory and immune response
000473	hsa-miR-150-5p	UCUCCCAACCCUUGUACCAGUG	Inflammatory and immune response/BBB permeability
001191	hsa-miR-153-3p^b^	UUGCAUAGUCACAAAAGUGAUC	Neurogenesis
002623	hsa-miR-155-5p^b^	UUAAUGCUAAUCGUGAUAGGGGU	Inflammatory and immune response/arteriogenesis
000389	hsa-miR-15a-5p^b^	UAGCAGCACAUAAUGGUUUGUG	Vascular angiogenesis
000390	hsa-miR-15b-5p^b^	UAGCAGCACAUCAUGGUUUACA	Apoptosis/inflammatory response
000391	hsa-miR-16-5p^b^	UAGCAGCACGUAAAUAUUGGCG	Vascular angiogenesis
002308	hsa-miR-17-5p^b^	CAAAGUGCUUACAGUGCAGGUAG	Neovascularization/apoptosis/proliferation
000480	hsa-miR-181a-5p^b^	AACAUUCAACGCUGUCGGUGAGU	Apoptosis/inflammatory response
000482	hsa-miR-181c-5p^b^	AACAUUCAACCUGUCGGUGAGU	Apoptosis/inflammatory response
000494	hsa-miR-195-5p^b^	UAGCAGCACAGAAAUAUUGGC	Homeostasis of vessel smooth muscle cells
000396	hsa-miR-19b-3p^b^	UGUGCAAAUCCAUGCAAAACUGA	Neovascularization/apoptosis/proliferation
000508	hsa-miR-204-5p^b,c^	UUCCCUUUGUCAUCCUAUGCCU	Apoptosis/proliferation
000580	hsa-miR-20a-5p^b^	UAAAGUGCUUAUAGUGCAGGUAG	Neovascularization/apoptosis/proliferation
000397	hsa-miR-21-5p^b^	UAGCUUAUCAGACUGAUGUUGA	Apoptosis/inflammatory
000524	hsa-miR-221-3p^b^	AGCUACAUUGUCUGCUGGGUUUC	Apoptosis/inflammatory
002295	hsa-miR-223-3p^b^	UGUCAGUUUGUCAAAUACCCCA	Inflammatory and immune response
000399	hsa-miR-23a-3p	AUCACAUUGCCAGGGAUUUCC	Mitochondrial function/apoptosis
000400	hsa-miR-23b-3p^b^	AUCACAUUGCCAGGGAUUACC	Mitochondrial function/apoptosis
000402	hsa-miR-24-3p^b^	UGGCUCAGUUCAGCAGGAACAG	Apoptosis/proliferation
000408	hsa-miR-27a-3p^b^	UUCACAGUGGCUAAGUUCCGC	Autophagy/apoptosis
000409	hsa-miR-27b-3p^b^	UUCACAGUGGCUAAGUUCUGC	Autophagy/apoptosis
002112	hsa-miR-29a-3p^b^	UAGCACCAUCUGAAAUCGGUUA	Apoptosis/proliferation/immune response
000413	hsa-miR-29b-3p^b^	UAGCACCAUUUGAAAUCAGUGUU	Apoptosis/proliferation/immune response
000587	hsa-miR-29c-3p^b^	UAGCACCAUUUGAAAUCGGUUA	Apoptosis/proliferation/immune response
000426	hsa-miR-34a-5p^b^	UGGCAGUGUCUUAGCUGGUUGU	Autophagy/inflammatory response
002102	hsa-miR-34b-3p^b^	CAAUCACUAACUCCACUGCCAU	Autophagy/inflammatory response
001043	hsa-miR-497-5p	CAGCAGCACACUGUGGUUUGU	Apoptosis/inflammatory response
002268	hsa-miR-874-3p	CUGCCCUGGCCCGAGGGACCGA	Apoptosis/inflammatory response
000583	hsa-miR-9-5p^b^	UCUUUGGUUAUCUAGCUGUAUGA	Neurogenesis and differentiation
000430	hsa-miR-92a-3p^b^	UAUUGCACUUGUCCCGGCCUG	Neovascularization/apoptosis/proliferation
001182	hsa-miR-124-3p^b^	UAAGGCACGCGGUGAAUGCC	Neurogenesis, differentiation, and inflammatory response
002571	mmu-miR-155-5p^d^	UUAAUGCUAAUUGUGAUAGGGGU	
001141	hsa-miR-451a^b^	AAACCGUUACCAUUACUGAGUU	Apoptosis/inflammatory response/RBC enriched

**^*a*^All selected miRNAs are known to be associated with CNS injuries or cerebral vasculature damage. ^*b*^miRNA has been implicated in biomarker studies related to CNS (TBI or SCI) or stroke. ^*c*^miRNA is a potential normalizer for CSF. ^*d*^mmu-miR-155-5p was included for a separate unrelated rodent study.**

**FIGURE 2:**
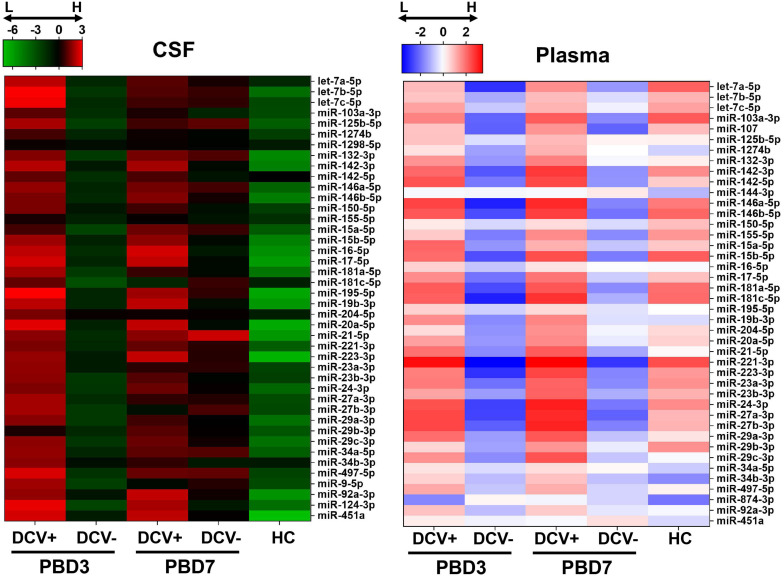
Heatmaps of overall miRNA expression in CSF and plasma specimens collected from aSAH patients and healthy controls (HC). Normalized –ΔC_RT_ was used to generate the mean expression for each miRNA in each group.

A miRNA was deemed detectable if it exhibited a C_rt_ < 35 in at least two-thirds of the total specimens. Based on these criteria, six miRNAs from the CSF samples (miR-107, miR-144-3p, miR-153-3p, miR-15a-5p, miR-29b-3p, and miR-874-3p) and six miRNAs from the plasma samples (miR-124-3p, miR-144-3p, miR-1298-5p, miR-153-3p, miR-874-3p, and miR-9-5p) were removed from further analysis. Overall, a clear and distinct miRNA expression pattern was observed between the DCV+ and DCV- groups, aSAH and HC groups, and between sampling times (PBD3 vs. PBD7) in both biofluids (Figure 2).

### Individual miRNA Performance in CSF and Plasma

**FIGURE 3:**
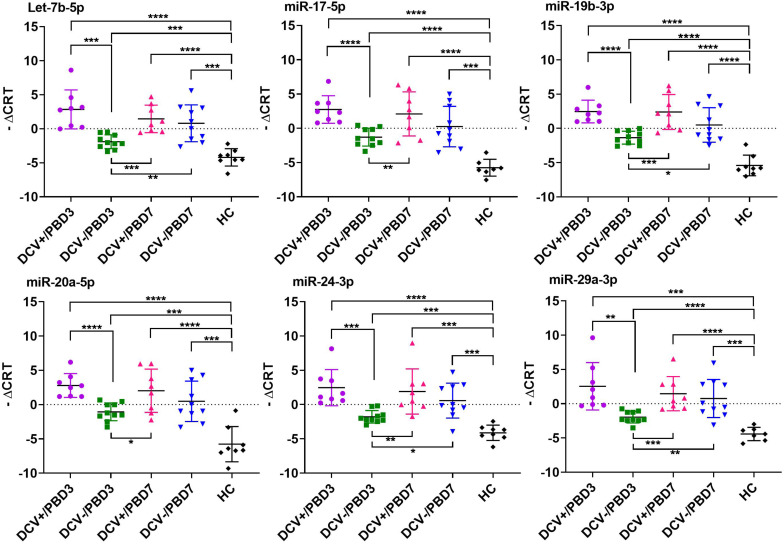
Individual CSF miRNAs that showed highly differential expression in DCV+, DCV-, and HC groups. The differential miRNA expression levels are expressed as –ΔC_RT_ and a two tailed Student’s *t*-test was used to evaluate differences between groups. Significant levels expressed as: ****p < 0.0001; ***p = 0.0001–0.001; **p = 0.001–0.01; *p = 0.01–0.05.

**FIGURE 4:**
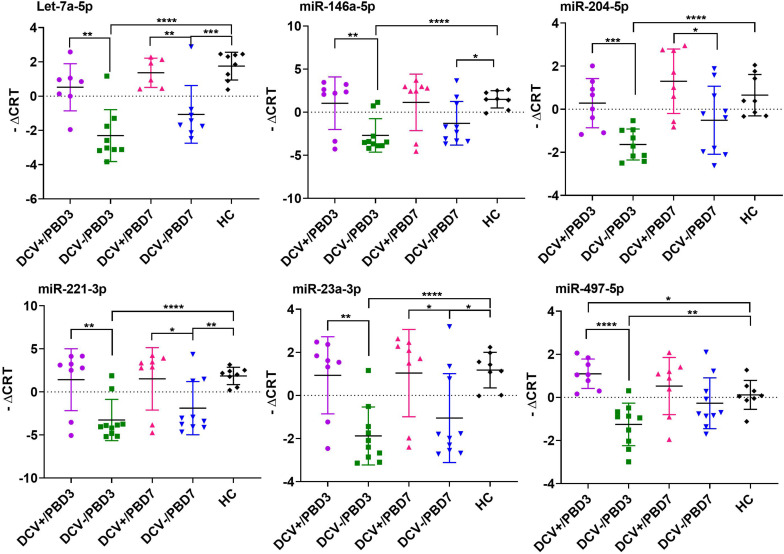
Individual plasma miRNAs that showed highly differential expression in DCV+, DCV–, and HC groups. The differential miRNA expression levels are expressed as –ΔC_RT_ and a two tailed Student’s *t*-test was used to evaluate the difference between groups. Significant levels expressed as: ****p < 0.0001; ***p = 0.0001–0.001; **p = 0.001–0.01; *p = 0.01–0.05.

The majority of the selected miRNAs in CSF and plasma showed significant differential expression between DCV+ and DCV- at PBD3, but was reduced by PBD7 (Table 3). The difference was especially robust in CSF specimens obtained at PBD3. Specifically, 37 miRNAs from the CSF demonstrated highly significant differences in samples collected from DCV+ relative to DCV- patients at PBD3. Several miRNAs including Let-7b-5p, miR-15b-5p, miR-17-5p, miR-19b-3p, miR-20a-5p, miR-24-3p, and miR-29a-3p exhibited remarkable differential levels between the DCV+ and DCV- groups at PBD3, as well as between the DCV+/PBD3 group and HCs (Figure 3 and Supplementary Figure 2). With respect to the plasma specimens, 29 miRNAs demonstrated significant differential expression between the DCV+ and DCV- groups at PBD3 (Table 3), with several miRNAs (Let-7a-5p, miR-146a-5p, miR-204-5p, miR-221-3p, miR-23a-3p, miR-497-5p) showing high differential expression patterns (Figure 4).

**TABLE 3:** Significant differential expression levels of brain and vasculature injury related miRNAs.

**miRNA**	**CSF**						**Plasma**					
	**I**	**II**	**III**	**IV**	**V**	**VI**	**I**	**II**	**III**	**IV**	**V**	**VI**
let-7a-5p	**	ns	*	ns	ns	ns	**	**	ns	****	ns	***
let-7b-5p	***	ns	****	***	****	***	ns	ns	ns	*	ns	ns
let-7c-5p	**	ns	**	ns	*	*	ns	ns	ns	*	ns	ns
miR-103a-3p	*	ns	UD in heathy controls				ns	ns	ns	****	ns	**
miR-107	UD						***	*	***	ns	ns	*
miR-125b-5p	**	ns	**	ns	**	**	ns	ns	ns	ns	ns	ns
miR-1274b	*	ns	*	ns	**	**	**	ns	**	ns	**	ns
miR-1298-5p	ns	ns	ns	ns	**	ns	UD					
miR-132-3p	**	ns	**	**	***	**	*	ns	ns	**	ns	ns
miR-142-3p	***	ns	****	***	***	****	**	*	ns	***	ns	*
miR-142-5p	***	ns	UD in heathy controls				*	ns	ns	**	ns	ns
miR-144-3p	UD						UD					
miR-146a-5p	***	ns	****	***	****	***	**	ns	ns	****	ns	*
miR-146b-5p	**	ns	***	***	***	***	*	ns	ns	****	ns	**
miR-150-5p	**	ns	****	**	*	*	ns	ns	ns	**	ns	ns
miR-153-3p	UD						UD					
miR-155-5p	ns	ns	ns	ns	ns	ns	**	*	ns	****	ns	***
miR-15a-5p	UD						**	ns	ns	**	ns	*
miR-15b-5p	****	ns	***	*	**	*	*	ns	ns	***	ns	**
miR-16-5p	***	ns	****	***	**	**	ns	ns	ns	ns	ns	ns
miR-17-5p	***	ns	****	***	**	**	*	ns	ns	*	ns	ns
miR-181a-5p	***	ns	*	ns	*	ns	**	ns	ns	***	ns	**
miR-181c-5p	ns	ns	ns	ns	ns	ns	**	ns	ns	***	ns	*
miR-195-5p	***	ns	****	**	***	**	ns	ns	ns	ns	ns	ns
miR-19b-3p	****	ns	****	****	****	****	*	ns	ns	ns	ns	ns
miR-204-5p,	**	ns	****	*	*	ns	***	*	ns	****	ns	ns
miR-20a-5p	****	ns	****	***	****	***	*	ns	ns	*	ns	ns
miR-21-5p	**	ns	****	****	****	****	**	ns	ns	*	ns	ns
miR-221-3p	**	ns	**	*	***	***	**	*	ns	****	ns	**
miR-223-3p	***	ns	****	****	****	****	**	ns	ns	****	ns	ns
miR-23a-3p	**	ns	***	**	***	***	**	*	ns	****	ns	*
miR-23b-3p	*	ns	ns	ns	ns	ns	**	*	ns	***	ns	*
miR-24-3p	***	ns	****	***	***	***	**	ns	ns	***	ns	*
miR-27a-3p	***	ns	****	*	**	****	**	ns	ns	***	ns	*
miR-27b-3p	***	ns	**	ns	ns	*	*	ns	ns	***	ns	*
miR-29a-3p	**	ns	***	****	****	***	**	ns	ns	**	ns	ns
miR-29b-3p	UD							*	ns	ns	ns	ns
miR-29c-3p	**	ns	**	*	***	**	*	ns	ns	ns	ns	ns
miR-34a-5p	**	ns	***	***	****	****	*	ns	*	ns	*	*
miR-34b-3p	**	ns	***	ns	ns	ns	ns	ns	**	ns	**	ns
miR-497-5p	***	ns	**	ns	**	**	****	ns	*	**	ns	ns
miR-874-3p	UD						UD					
miR-9-5p	*	ns	ns	ns	ns	ns	UD					
miR-92a-3p	***	ns	****	****	****	****		ns	ns	ns	ns	ns
nsmiR-124-3p	**	ns	**	*	**	*	UD					
miR-451a	***	ns	*	ns	ns	ns	ns	ns	ns	ns	ns	ns

Custom brain and vasculature injury miRNA panel TLDA was used to analyze CSF and plasma specimens from Group A patients (8× DCV+, 10× DCV-, 8× HC). The differential expression was evaluated using Student’s t-test (2-tailed) between I: DCV+ vs. DCV-, PBD3; II: DCV+ vs. DCV-, PBD7; III: DCV+ vs. HC, PBD3; IV: DCV- vs. HC, PBD3; V: DCV+ vs. HC, PBD7; VI: DCV- vs. HC, PBD7. Asterisk symbols for significant levels: ****p < 0.0001; ***p = 0.0001–0.001; **p = 0.001–0.01; *p = 0.01–0.05; ns: p ≥ 0.05. UD, undetectable.

### Receiver Operating Characteristic (ROC) Curves of Individual miRNAs

**FIGURE 5:**
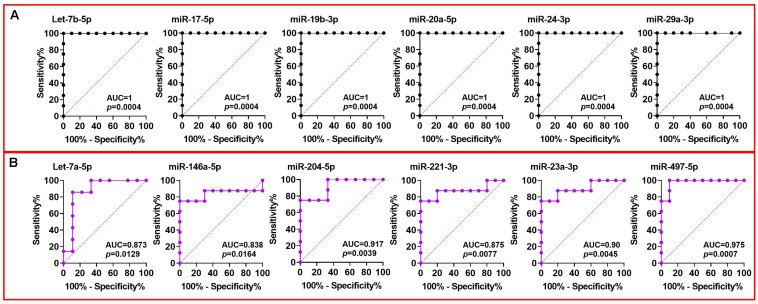
ROC analysis of miRNAs using Group A CSF and plasma data collected at PBD3 from aSAH patients. ROC curve constructed using **(A)** DCV+ and DCV– groups of CSF miRNA data; **(B)** DCV+ and DCV– groups of plasma miRNA data.

The sensitivity and specificity of individual miRNAs as predictors of DCV were examined using the area under the ROC curve (AUC). Many miRNAs demonstrated an AUC greater than 0.9 in CSF collected at PBD3 indicating a highly significant predictive ability of these miRNAs (Figure 5A). Of note, the AUC of Let-7b-5p, miR-15b-5p, miR-17-5p, miR-19b-3p, miR-20a-5p, miR-24-3p, and miR-29a-3p reached 1 (AUC = 1), signifying a perfect discrimination between DCV+ and DCV- at a time point prior to DCV onset. Similarly, several plasma miRNAs achieved AUCs greater than 0.8 (Figure 5B), demonstrating a robust ability of selected miRNA for discriminating DCV in two biofluids.

High AUC values were also achieved when comparing aSAH and HC using CSF/PBD3 data (Supplementary Figure 3), indicating that selected CSF miRNAs can also discriminate aSAH patients from HCs.

### Prediction Accuracy of the Custom miRNA Panel on Group B and Group C

The CSF/PBD3 data from Group A was then used to build a predicting model, and the datasets from the remaining 15 cases from Group B (including 2 replicates from Group A) and Group C were used for retrospective risk prediction. A risk prediction accuracy of 93% was achieved with miR-19b-3p and miR-29a-3p using the corresponding cutoff values of -0.481 and 0.53, respectively (Table 4 and Supplementary File 1). In contrast, miR-24-3p had only 73% accuracy using the corresponding cutoff value of -0.011.

**TABLE 4:** Overall performance of DCV risk prediction of the custom miRNA panel.

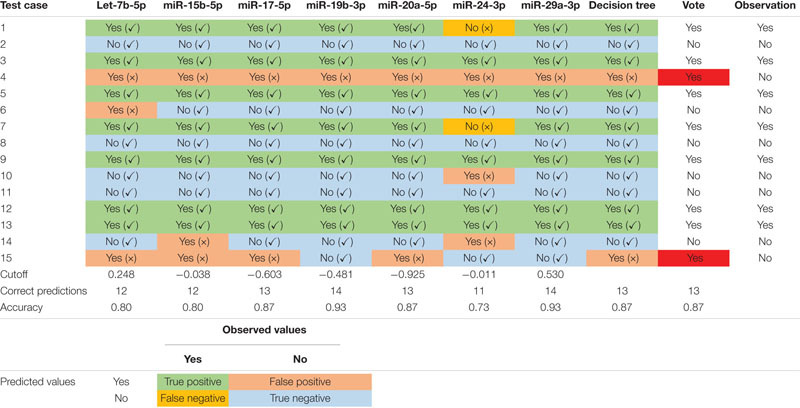

**The table includes seven highly predictive individual miRNAs and the prediction using the decision tree. The cutoff values for each individual miRNA predictor were the mean of the values from the DCV+ and DCV- cases. The cutoff value calculations and corresponding specificity and sensitivity in this prediction can be found in Supplementary File 1.**

#### Decision Tree (DT) Results

**FIGURE 6:**
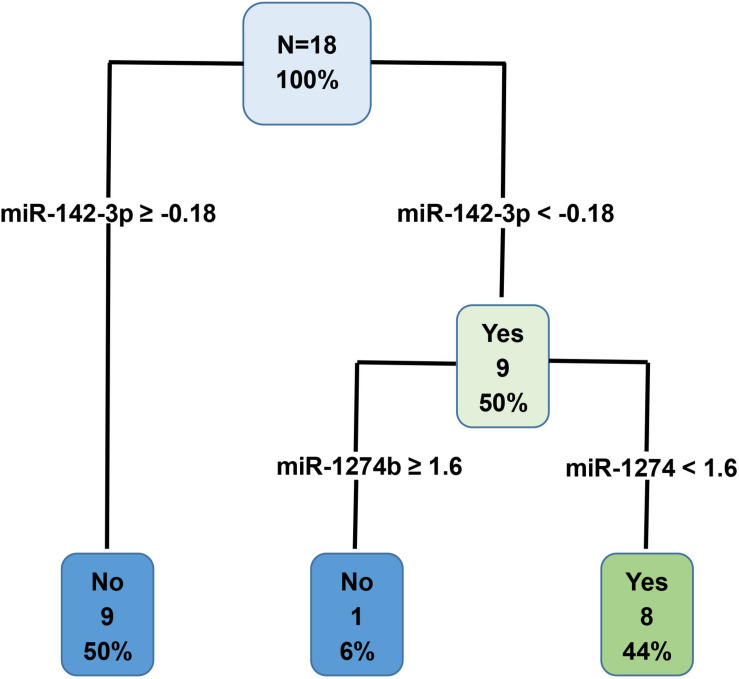
Decision tree prediction model was generated using the Group A training dataset. Out of the 19 variables entered into the model, miR-142-3p and miR-1274b were identified as key variables in the prediction process. Specifically, these two miRNAs correctly predicted 100% of the Group A training dataset.

MiRNAs with an AUC less than 1 were used to generate a decision tree model (Figure 6). Out of the 19 miRNAs entered into the model, miR-142-3p and miR-1274b were identified as key variables in the prediction process. This model correctly predicted 100% of the training dataset (Group A, Figure 6), and 87% of the test dataset (Group B + Group C, Table 4).

#### Overall Prediction of the miRNA Panel

Table 4 presents a consensus prediction table incorporating 7 miRNAs with a perfect AUC of 1 and additional 19 miRNAs from the DT. The prediction of each testing subject was determined when at least five of the eight criteria were met for either DCV+ or DCV-. Notably, the majority of the predictions were uniformly accurate across the test dataset with exceptions in only two out of 15 test cases. Case 4 had only false positive predictions, while case 15 resulted in 5 out of 8 false positive predictions. Despite the two false positive predictions, the overall performance of the custom panel in predicting DCV risk was 87% accurate.

## Discussion

The potential of miRNAs serving as biomarkers for aSAH complications was recently explored in several reports (Su et al., 2015; Powers et al., 2016; Bache et al., 2017; Kikkawa et al., 2017; Lu et al., 2017; Stylli et al., 2017; Lopes et al., 2018; Pulcrano-Nicolas et al., 2018; Sheng et al., 2018a). These studies confirmed that dysregulated miRNA expression is associated with aSAH and its acute complications, which could be observed in extracellular fluids. However, there is no consensus in the limited literature for targeting biofluid miRNAs as a valid biomarkers for predicting DCV. This is due, in part, to significant variability across studies, including differences in sampling size (4–129 cases), type of biofluid used (whole blood, CSF, serum, plasma), collection time points (1–14 days), as well as miRNA analysis methodologies. Several studies utilized time-consuming and costly analytical methodologies such as next-generation sequencing and microarray (Su et al., 2015; Kikkawa et al., 2017; Pulcrano-Nicolas et al., 2018) that, while informative, may limit clinical applications.

The major difference between our study and other reports is that we employed a panel of selected miRNAs that is highly relevant to brain and vasculature injury events. This strategy directly targets a condition with a dedicated set of miRNAs involving related pathological processes. By doing so, the likelihood of identifying responsive biomarkers is much greater and may be achieved using a smaller patient cohort. Furthermore, multiple miRNAs can be used concomitantly in the overall assessment of DCV risk to increase prediction accuracy.

Our study revealed a robust differential expression of the selected miRNAs between DCV+ and DCV- patients, as well as between aSAH and HCs in both CSF and plasma, with the CSF samples showing the most pronounced difference. This differential pattern resulted in a high prediction accuracy of 87% in our independent cohort studies. It is important to point out that all of the predictor miRNAs falsely identified case 4 to be DCV positive (Table 4), and five of the eight predictors incorrectly identified case 15 to be DCV positive. It is not entirely clear why these two cases resulted in incorrect predictions, and it may be related, in part, with the complex clinical conditions or comorbidities of the individuals.

Several miRNAs in our panel were previously reported to link to aSAH related complications. Significantly increased levels of miR-21-5p and miR-221-3p in CSF were shown to associate with delayed cerebral ischemia (DCI) and miR-221-3p, miR-132-3p, and miR-19b-3p in CSF were associated with DCV (Bache et al., 2017). In addition, CSF expression levels of miR-27a-3p were differentially expressed between aSAH patients with or without DCV (Stylli et al., 2017). Finally, temporal changes in Let-7b-5p and miR-92a-3p in CSF, and miR-15a in both CSF and plasma of DCI patients have been reported (Powers et al., 2016; Kikkawa et al., 2017). In other studies, the levels of miR-146a-5p, miR-17-5p, and miR-451a in whole blood were significantly different in aSAH patients compared to the HCs (Lopes et al., 2018). Su et al. (2015) reported that miR-132-3p is differentially expressed in peripheral blood between DCI+ and DCI- patients. Our data were generally consistent with the findings of these prior studies. However, because the wide differences in the patient cohorts, specimens used, sampling/analysis time points, detection methodologies, and variations on study focus (e.g., aSAH vs. control, occurrence of DCV or DCI, etc.), it is difficult to make direct comparisons between our results and those reported in the previously published studies.

Another important advantage of our panel is that individual miRNAs are easily replaced if they do not perform well. Not all of the miRNAs on our current panel contributed to DCV prediction and several miRNAs were excluded from further analysis due to their low expression levels (e.g., miR-144-5p, miR-153-3p, and miR-874-3p). In addition, other miRNAs, such as miR-1298-5p in CSF and miR-16-5p in plasma, did not differentiate between DCV+ and DCV-. Therefore, replacing these miRNAs with other candidate miRNAs, for example, miR-502, miR-516a-5p, and miR-1297 (Lai et al., 2017; Stylli et al., 2017), that may prove more effective in DCV prediction will be considered in future studies.

We recognize that there are several limitations with our current study. First, the patient cohort was relatively small, and samples were limited to two institutions. This can be partially attributed to the rarity of aSAH. The incidence of aSAH is 6–16 cases per 100,000 persons, with approximately 30,000 cases occurring each year in the United States (de Rooij et al., 2007). To fully address this limitation and to validate the DCV miRNA panel, future studies will need to expand the patient cohort and include recruitment from various medical institutions. In addition, we are currently exploring the possibility of validating our data in aSAH animal models. Second, there were only two sampling time points. Prior studies reported that altered miRNA expression levels in biofluids following aSAH are temporally dynamic (Powers et al., 2016; Kikkawa et al., 2017; Sheng et al., 2018b; Bache et al., 2020). Therefore, it will be critical to identify a time point that shows the greatest differences in miRNA expression levels and, more importantly, provides the earliest prediction to allow clinicians time to make medical decisions. Third, the detection of DCV miRNAs was solely based on TaqMan miRNA assay technology. Although this technology has also been successfully applied in other aSAH miRNA studies (Bache et al., 2017, 2020), it will be important to validate this miRNA panel using a different detection method to avoid technological bias. Lastly, the current study did not correlate the expression of selected miRNAs with other aSAH complications, such as DCI or early brain injury, nor with the prognosis. Our focus was to examine DCV after aSAH and future studies are warranted to evaluate how this novel miRNA biomarker panel might be applied these other scenarios and how it might impact clinical practice.

## Data Availability Statement

The data presented in the study are deposited in the National Center for Biotechnology Information (NCBI) Gene Expression Omnibus (GEO) repository (https://www.ncbi.nlm.nih.gov/geo/), accession number GSE165608.

## Ethics Statement

The studies involving human participants were reviewed and approved by the University of Kentucky Institutional Review Board. The patients/participants provided their written informed consent to participate in this study.

## Author Contributions

WXW, KWH, and JES: project concept and design and manuscript preparation. WXW and KWH: experimental implementations and data acquisition and analysis. KX, DWF, and WXW: statistical analysis. KWH, JES, and WXW: specimen procurement. WXW, KWH, JES, KX, and DWF: manuscript editing and review. All authors contributed to the article and approved the submitted version.

## Conflict of Interest

The authors declare that the research was conducted in the absence of any commercial or financial relationships that could be construed as a potential conflict of interest.

## Supplementary Material

Supplementary Figure 1 Workflow and estimated time needed for miRNA isolation and TaqMan TLDA analysis.

Supplementary Figure 2 Distribution of miRNA expression values for 26 miRNAs between DCV+ and DCV- of the Group A cases. Each filled circle corresponds to a single case with “Yes” referring to DCV+ and “No” referring to DCV-.

Supplementary Figure 3 ROC curves constructed using CSF miRNA data (PBD3) from aSAHs (DCV+ plus DCV- group) and HCs.

Supplementary File 1 The file contains (1) variables without missing data; (2) DCV prediction cutoff value; (3) CSF dataset; (4) plasma dataset.
