# Supplementary figures and images for: A Highly Predictive MicroRNA Panel for Determining Delayed Cerebral Vasospasm Risk Following Aneurysmal Subarachnoid Hemorrhage
Source: Front Mol Biosci. 2021 May 14;8:657258. doi: 10.3389/fmolb.2021.657258 (PMC8163224; doi:10.3389/fmolb.2021.657258)

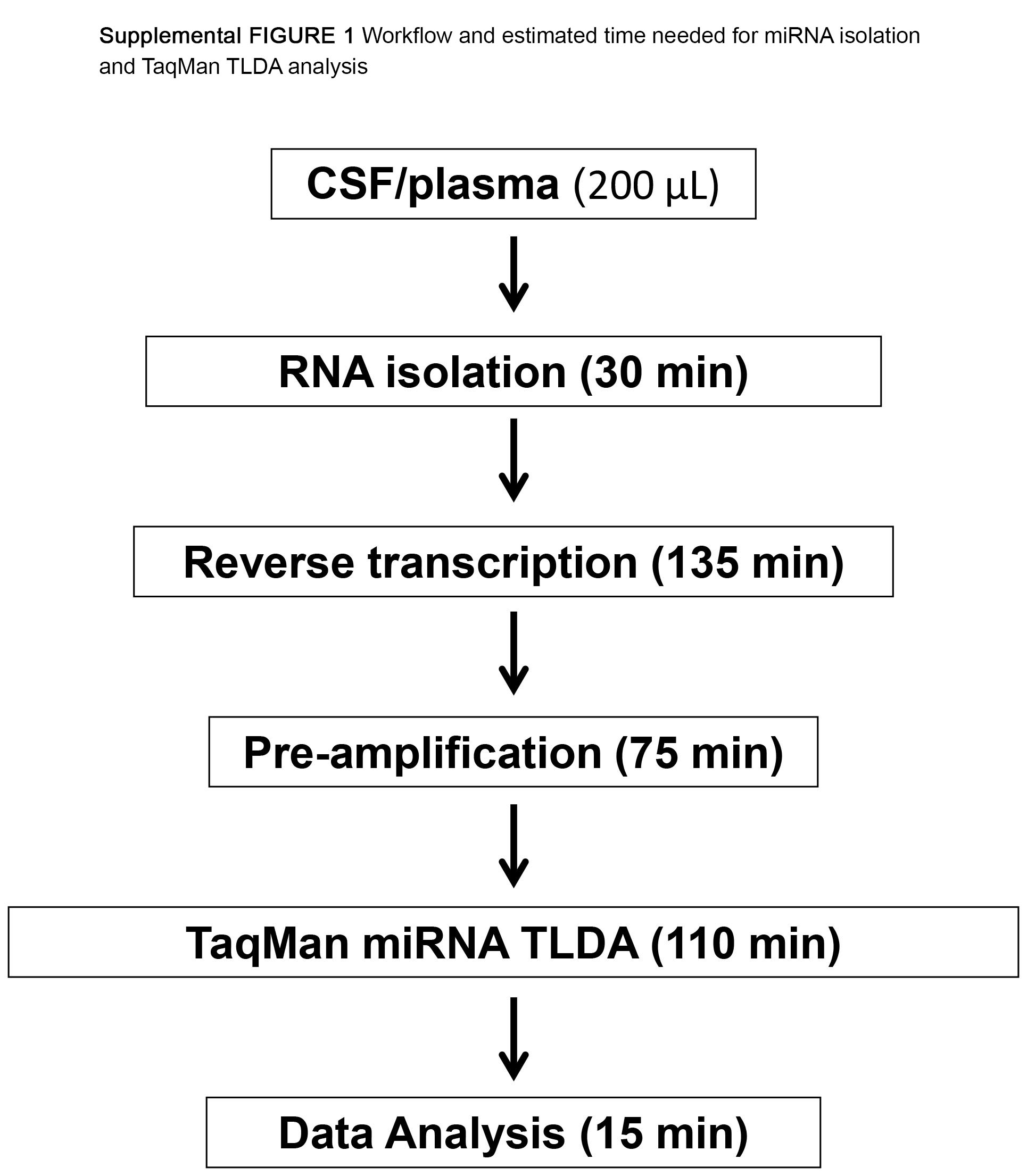

Supplement: Supplementary Figure 1 — Workflow and estimated time needed for miRNA isolation and TaqMan TLDA analysis. [file Image_1.jpg]

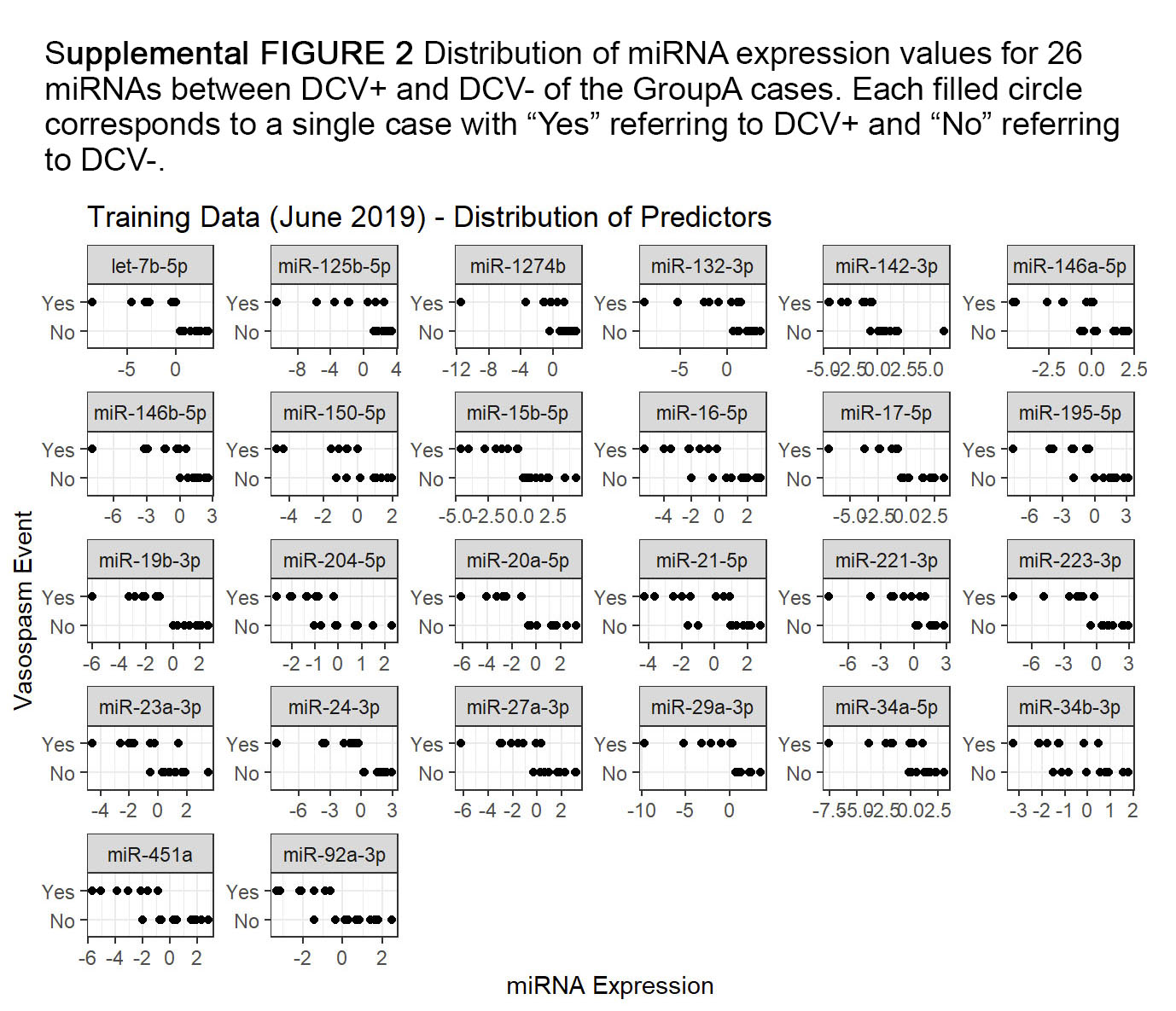

Supplement: Supplementary Figure 2 — Distribution of miRNA expression values for 26 miRNAs between DCV+ and DCV- of the Group A cases. Each filled circle corresponds to a single case with “Yes” referring to DCV+ and “No” referring to DCV-. [file Image_2.jpg]

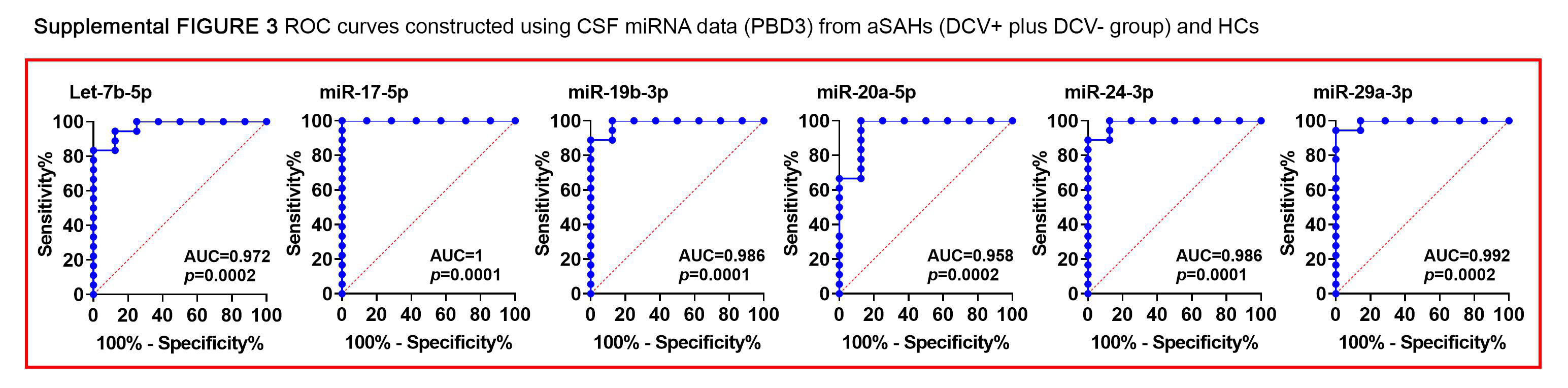

Supplement: Supplementary Figure 3 — ROC curves constructed using CSF miRNA data (PBD3) from aSAHs (DCV+ plus DCV- group) and HCs. [file Image_3.jpg]
